# Phase II study of sequential chemotherapy with docetaxel–estramustine followed by mitoxantrone–prednisone in patients with advanced hormone-refractory prostate cancer

**DOI:** 10.1038/sj.bjc.6604090

**Published:** 2007-11-20

**Authors:** L Galli, A Fontana, C Galli, L Landi, E Fontana, A Antonuzzo, M Andreuccetti, E Aitini, R Barbieri, R Di Marsico, A Falcone

**Affiliations:** 1 Division of Medical Oncology, Azienda USL 6 of Livorno, Livorno 57100, Italy; 2 Division of Medical Oncology and Hematology, Azienda Ospedaliera C Poma Mantova, Italy; 3 Department of Oncology Transplants and New Technologies in Medicine, University of Pisa, Italy

**Keywords:** prostate cancer, docetaxel, estramustine, mitoxantrone, sequential chemotherapy

## Abstract

Sequential chemotherapy may improve treatment efficacy avoiding the additive toxicity associated with concomitant polichemotherapy in hormone-refractory prostate cancer (HRPC). Forty patients received docetaxel 30 mg m^−2^ intravenous (i.v.), weekly, plus estramustine 280 mg twice daily for 12 weeks. After 2 weeks rest, patients with a decline or stable PSA were treated with mitoxantrone 12 mg m^−2^ i.v. every 3 weeks plus prednisone 5 mg twice daily for 12 cycles. Forty patients were assessable for toxicity after docetaxel/estramustine. Main toxicities were grade 3–4 AST/ALT or bilirubin increase in seven patients (17.5%) and deep venous thrombosis (DVT) in four patients (10%). Twenty-seven patients received mitoxantrone/prednisone. Main toxicities included DVT in one patient (3.7%) and congestive heart failure in two patients (7%). Thirty-nine patients were assessable for PSA response. Twenty-nine patients (72.5%; 95% CI 63–82%) obtained a ⩾50% PSA decline with 15 patients (37.5%; 95% CI 20–50%) that demonstrated a ⩾90% decrease. Median progression-free and overall survival were respectively 7.0 (95% CI 5.8–8.2 months) and 19.2 months (95% CI 13.9–24.3 months). In conclusion, although this regimen demonstrated a favourable toxicity profile, sequential administration of mitoxantrone is not able to improve docetaxel activity in patients with HRPC.

Prostate cancer is the most common cancer in men and the third estimated cause of death of cancer in the United States in 2006 (Jemal *et al*, 2006). Androgen ablation is the standard initial management for metastatic prostate cancer; however, all patients develop hormone-refractory disease after a median time of 18–24 months. In such condition, second-line hormonal agents lead to low response rates and brief disease control and multidisciplinary treatment includes symptomatic treatments, bisphosphonates, radiotherapy, radioisotopes and chemotherapy (Pienta and Smith, 2005).

Two randomised phase III trials of mitoxantrone plus corticosteroids *vs* corticosteroids alone (Tannock *et al*, 1996; Kantoff *et al*, 1999) demonstrated an improvement in quality of life, pain relief and PSA response rate in favour of mitoxantrone regimens. Although this significant palliative activity was not matched with an improvement in survival, mitoxantrone plus prednisone was considered the standard chemotherapy regimen for hormone-refractory prostate cancer (HRPC) for a long time. New chemotherapy agents such as docetaxel combined with estramustine showed interesting activity in phase II trials. They shared similar mechanisms of action (microtubule stabilisation and mitotic process disruption) and showed synergistic activity in prostate cancer cell lines (Stearns *et al*, 1985; Kreis *et al*, 1997). Phase II studies of single agent docetaxel, given every 3 weeks or as a weekly schedule, showed a PSA response rate of about 45% and an interesting objective response rate, particularly for the weekly regimen (40%) (Picus and Schultz, 1999; Beer *et al*, 2001). Combination of docetaxel with estramustine led to a very promising activity. A PSA response rate between 45 and 68% with an objective response rate of 20–55% was observed in every 3 weeks docetaxel plus estramustine phase II trials (Petrylak *et al*, 2000; Savarese *et al*, 2001; Sitki Copur *et al*, 2001; Sinibaldi *et al*, 2002). Moreover, weekly docetaxel plus estramustine produced a ⩾50% decline in PSA in 76% of patients and an objective response rate of 58% without grade 4 toxicity (Sitki Copur *et al*, 2001). Recently, two randomised phase III trials have demonstrated a significant improvement in overall survival (OS), pain control, quality of life and PSA response, for docetaxel-based chemotherapy, which has become the standard treatment for metastatic HRPC (Petrylak *et al*, 2004; Tannock *et al*, 2004). However, no second-line chemotherapy is currently available for HRPC, and mitoxantrone has demonstrated a PSA response rate of 12–20% even in taxane-resistant patients showing a noncomplete cross resistance between docetaxel and mitoxantrone (Michels *et al*, 2006; Rosenberg *et al*, 2006). In the attempt to improve treatment activity, combination of three (Colleoni *et al*, 1997; Smith *et al*, 1999; Segawa *et al*, 2005) or more (Smith *et al*, 2003) chemotherapy agents was investigated in phase II studies. These trials reported a PSA response rate in 58–65% of patients and a measurable disease response between 32 and 58%, but an incidence of grade 3–4 haematological toxicity in 12–48% of patients. Moreover, febrile neutropaenia in 15% of patient and one toxic death were reported in one study (Colleoni *et al*, 1997). The significant toxicity observed with polichemotherapy regimens makes the combination of docetaxel plus estramustine and mitoxantrone probably not easily feasible, particularly in this often elderly and ‘fragile’ population of patients. At the time of study design, no data concerning docetaxel efficacy or better schedule of administration were available. We design this sequential phase II study of 12 weeks docetaxel regimen in accordance to previous findings by Beer *et al* (2001) and Sitki Copur *et al* (2001). Moreover, we administered sequential mitoxantrone/prednisone in case of response of stable disease to expose patients, who mostly benefit from docetaxel chemotherapy, to both agents at therapeutic doses, avoiding additive toxicity and potentially increasing activity.

## MATERIALS AND METHODS

### Eligibility criteria

Eligible patients had histologically documented adenocarcinoma of the prostate and had failed to benefit from androgen ablation and subsequent antiandrogen withdrawal for their metastatic or locally advanced disease. All patients met the following criteria: age ⩾18 years; measurable disease progression or PSA serum level increasing on three consecutive measurements at least 2 weeks apart; PSA ⩾10 ng ml^−1^; Eastern Cooperative Oncology Group (ECOG) performance status ⩽2; no prior chemotherapy included estramustine; discontinued prior antiandrogen treatment by at least 4 weeks (6 weeks in the case of bicalutamide); adequate bone marrow function (leucocytes ⩾3500 ml^−1^, neutrophil count ⩾1500 ml^−1^, haemoglobin level ⩾10 g per 100 ml, platelets ⩾100 000 ml^−1^); adequate liver function (total serum bilirubin level <1.5 mg per 100 ml, AST and ALT <2.5 upper normal limit); adequate renal function (serum creatinine level <1.5 mg per 100 ml); and written informed consent. Patients with uncontrolled metabolic diseases, cardiovascular disease (uncontrolled arrhythmia, myocardial infarction within 2 years before enrolment, unstable angina, NYHA grade II or greater congestive heart failure) or active infections were excluded.

### Treatment

All eligible patients received docetaxel 30 mg m^−2^ by 1 h intravenous (i.v.) infusion on day 1 on a weekly schedule plus estramustine phosphate 280 mg orally two times daily from day 1, for 12 weeks. After 2 weeks rest from the last docetaxel infusion, patients who had achieved ⩾50% PSA serum value decline or had stable disease received mitoxantrone 12 mg m^−2^ by 1 h i.v. infusion every 3 weeks plus prednisone 5 mg orally two times daily, continuously, up to 12 courses. Each chemotherapy sequence was discontinued in case of unacceptable toxicities or disease progression. Chemotherapy was withheld for 1 week in case of absolute neutrophil count <1000 ml^−1^ or if an NCI-CTC grade 2 or greater mucositis or gastrointestinal or cutaneous toxicities occurred. In the absence of recovery from toxicities within 3 weeks, patients discontinued the study. A 25% dose reduction for neutropaenic fever or any grade 3 toxicity and a 50% dose reduction for any grade 4 toxicity was recommended (except for grade 3–4 anaemia, alopecia, nausea and vomiting). Estramustine was interrupted for any grade 2–3 toxicity and restarted at a 50% dose reduction after recovery and was definitively stopped in case of any grade 4 toxicity. The LHRH analogue previously administered was continued during the study.

### Evaluation

Pretreatment evaluations included medical history, physical examination, complete blood count, serum biochemistry, PSA levels and pain evaluation according to the visual analogical scale (VAS). During docetaxel–estramustine treatment, the following were evaluated every week—physical examination, toxicity (NCI-CTC) record, blood count and serum biochemistry (creatinine, total bilirubin, AST, ALT, alkaline phosphatase); every 2 weeks—serum PSA level; every 3 weeks—blood count, total serum biochemistry and pain evaluations (VAS). During mitoxantrone–prednisone therapy, serum PSA level, blood count, total serum biochemistry and pain control (VAS) were evaluated every 3 weeks. Nuclear ventriculography (multiple-gated acquisition scanning, MUGASCAN) was performed before mitoxantrone administration and repeated at the sixth and ninth cycle.

### End points and response evaluation

The primary end point of the study was PSA response defined by a decrease ⩾50% from baseline, maintained for at least 4 weeks, in accordance with the consensus guidelines of the PSA Working Group (Bubley *et al*, 1999). PSA stabilisation was defined by a decrease <50% or an increase <25% from baseline, maintained for at least 4 weeks and progressive disease by an increase in serum PSA ⩾25% from baseline or ⩾50% from nadir, confirmed by two consecutive measurements at 2 weeks interval. Secondary end points were objective response rate according to RECIST criteria, toxicity (NCI-CTC), duration of PSA response, time to PSA progression, OS and pain control evaluations (VAS).

### Statistical analyses

This was a multicentre, prospective, nonrandomised phase II clinical trial where PSA response was the primary end point. According to the Simon's minimax two-stage design with *P*_0_=50%, *P*_1_=70%, *α*=0.10 and *β*=0.10, the enrolment of 23 patients was required in the first step of the study. If at least 12 objective responses were observed, a total of 39 assessable patients were enrolled. Study treatment was considered interesting if at least 24 out of 39 patients responded. Time to progression and OS were calculated from the date of first chemotherapy infusion to the date of progression or death/lost on follow-up, respectively. Time to progression and OS were analysed according to the Kaplan–Maier method. Response duration was calculated from the time of first objective response to the time of disease progression. An intent to treat analysis was performed.

## RESULTS

### Characteristics of the patients and treatment

Between September 2002 and July 2005, 40 patients from two institutions were enrolled. Median age was 72 years (range: 55–83), 19 patients (47.5%) had an ECOG performance status of 0 and 21 patients (52.5%) had an ECOG performance status of 1, median serum PSA level was 58 ng ml^−1^ (range: 12–912 ng ml^−1^), bone was the most frequent metastatic site (68%), 17 patients had a measurable disease (43%), 13 patients (33%) had undergone previous prostatectomy and 13 patients (33%) had received previous radiotherapy for their primary tumour. Eleven patients (27.5%) had a VAS ⩾1 bone pain (Table 1). In total, 397 courses of weekly docetaxel plus estramustine and a total of 199 three-weekly cycles of mitoxantrone plus prednisone were delivered. The median number of cycles per patient was 11.5 (range: 2–12) for the first part of the treatment and 7 (range: 2–12) for the second.

### Toxicity

Docetaxel plus estramustine followed by mitoxantrone plus prednisone were overall well-tolerated. All patients (40) were assessable for toxicity after docetaxel/estramustine. Only one patient developed NCI-CTC grade 2 neutropaenia and anaemia plus grade 3 thrombocytopaenia and grade 4 stomatitis that required hospitalisation. Seven patients (17.5%) showed a grade 3 increase of AST/ALT and/or bilirubin serum levels, resolved in four patients, after treatment delay or dose reductions. Four patients (10%) developed deep venous thrombosis (DVT) requiring estramustine discontinuation. Twenty-seven out of 40 patients received mitoxantrone/prednisone and were assessable for toxicity. Thirteen patients did not receive this second part of the treatment due to progressive disease (five patients), severe liver toxicity unresolved after 3 weeks rest (three patients) or patient's refusal to continue (five patients). The main grade 3–4 toxicities were neutropaenia in two patients (7%) and onycholysis in three (11%) patients. One patient developed DVT and two patients (7%) discontinued mitoxantrone due to the onset of congestive heart failure resolved with medical treatments. No toxic-related deaths were observed in each sequence of the planned treatment (Table 2).

### Activity

**Figure 1:**
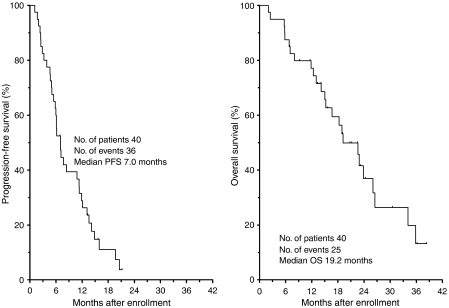
Progression-free and overall survival curves.

Thirty-nine patients were assessable for PSA response. One out of 40 patients refused study treatment continuation after the onset of an allergic reaction during docetaxel first administration. Overall, twenty-nine patients (72.5; 95% CI 63–82%) showed a confirmed PSA decrease ⩾50% from baseline, with 15 out of 29 patients (37.5; 95% CI 20–50%) demonstrating a confirmed ⩾90% decrease in PSA serum levels. Seven (17.5%) patients had a PSA stabilisation and three patients (7.5%), a PSA progression (Table 3). Seventeen patients had a measurable disease according to RECIST criteria. Two patients achieved a complete response (12%), six patients showed a partial response (35%) with an overall response rate of 47% and three patients showed a stable disease (17%). After docetaxel/estramustine, 24 patients with a PSA response and three patients with a PSA stabilisation received mitoxantrone plus prednisone. Four patients (10%), showing a ⩾50% PSA decrease with docetaxel plus estramustine treatment, improved their response achieving a PSA decrease ⩾90% during mitoxantrone/prednisone; no patients with PSA stabilisation during docetaxel/estramustine improved response with the second part of the treatment. Median duration of PSA response was 8.5 months (95% CI 3.85–13.10 months) in all study population and 9.4 months (95% CI 8–10.8 months) in responder patients who had received the two chemotherapy sequences. After a median follow-up of 24.2 months, median progression-free survival was 7.0 months (95% CI 5.8–8.2 months) and median OS was 19.2 months (95% CI 13.9–24.3 months) (Figure 1). Median progression-free survival and median OS in the 27 patients who had received the two chemotherapy sequences were 11.3 (95% CI 6.98–15.63 months) and 26.0 months (95% CI 20.6–31.4 months) respectively. Moreover, the principal patterns of disease progression, in the 27 patients who received all the chemotherapy sequence, was biochemical (20 patients, 74%), while skeletal or soft-tissue progression occurred in a minority of patients (four patients, 15% and two patients, 7% respectively). Eleven out of 40 patients (27.5%) had a basal VAS ⩾1 bone pain and among them, median baseline VAS was 4 (range: 1–10). Eight out of 11 patients (73%) experienced pain relief with the best response at week 4 of docetaxel/estramustine (median VAS value of 0, range: 0–4). Before receiving mitoxantrone/prednisone, 8 out of 27 patients (29.6%) had pain with a median VAS of 2.5 (range: 1–6). Pain relief was seen in three patients (37.5%) with best response after 1 cycle of mitoxantrone/prednisone (median VAS value of 2, range: 1–6).

## DISCUSSION

Until few years ago, chemotherapy for HRPC showed modest activity and no survival benefits and mitoxantrone plus prednisone was approved only for its palliative activity (Tannock *et al*, 1996; Kantoff *et al*, 1999). New chemotherapy regimens, such as docetaxel given every 3 weeks (Picus and Schultz, 1999) or as a weekly schedule (Beer *et al*, 2001), showed a PSA response rate of 46% (for both schedules) and an objective response rate of 24 and 40% respectively, in phase II studies. Moreover, its combination with estramustine demonstrated a very promising activity with manageable toxicity (Petrylak *et al*, 2000; Savarese *et al*, 2001; Sitki Copur *et al*, 2001; Sinibaldi *et al*, 2002). In particular, a randomised phase II trial of every 3 weeks or weekly docetaxel, in combination with estramustine and prednisone *vs* mitoxantrone and prednisone, reported a PSA response rate of 63%, a time to PSA progression of 9.3 months and a median survival of 18.4 months with the weekly schedule, which also presented a good toxicity profile (Oudard *et al*, 2005). Recently, two randomised phase III trials have demonstrated, for the first time, a statistically significant survival advantage for docetaxel-based regimens in patients with HRPC. Docetaxel given every 3 weeks plus prednisone, demonstrated an improvement in survival (median 18.9 *vs* 16.4 months, *P*=0.0009), pain control (RR: 35 *vs* 22%, *P*=0.01) and PSA response rate (45 *vs* 32%, *P*<0.001) when compared with mitoxantrone and prednisone (Tannock *et al*, 2004). The second study compared docetaxel and estramustine *vs* mitoxantrone and prednisone. A significant advantage was shown for the docetaxel-containing arm as concerning OS (median 17.5 *vs* 15.6 months, *P*=0.02), time to progression (median 6.3 *vs* 3.2 months, *P*<0.001) and PSA response rate (50 *vs* 27%, *P*<0.001) (Petrylak *et al*, 2004)^22^. Polichemotherapy regimens have also been evaluated in HRPC patients. Combination of three or more agents yielded a biochemical response rate of 58–65% and an objective response in 32–58% of patients (Colleoni *et al*, 1997; Smith *et al*, 1999, 2003; Segawa *et al*, 2005). However, such schedules showed grade 3–4 haematological toxicity ranging from 12 to 48% (Smith *et al*, 1999, 2003; Segawa *et al*, 2005) with febrile neutropaenia in 15% of patients and one toxic death reported in one study (Colleoni *et al*, 1997). To increase the activity, but to maintain a good toxicity profile, in an often elderly and unfit population we performed the present phase II trial of sequential chemotherapy with docetaxel plus estramustine, followed by mitoxantrone and prednisone. In particular, we selected the weekly docetaxel schedule for its better toxicity profile compared to the every 3 weeks schedule. Moreover, no complete cross resistance between docetaxel and mitoxantrone has been shown in HRPC and this observation further supported our sequential design. (Michels *et al*, 2006; Rosenberg *et al*, 2006). Our regimen was feasible with a favourable toxicity profile. After docetaxel/estramustine, there were no relevant grade 3–4 haematological toxicities with the exception of a grade 3 increase in serum levels of GOT, GPT and bilirubin in seven (17.5%) and DVT in four (10%) patients. Mitoxantrone/prednisone, after docetaxel/estramustine, was also well tolerated in docetaxel-pretreated patients. With regard to the activity, we have observed a confirmed PSA response rate of 72.5% (95% CI 63–82%) with a median duration of 8.5 months (95% CI 3.85–13.1 months) and an objective response rate of 47%. Median progression-free survival of 7.0 months (95% CI 5.8–8.2 months) and median OS of 19.2 months (95% CI 13.9–24.3 months) are also interesting (Figure 1). Sequential chemotherapy was, also, evaluated in a previous phase II study in HRPC. In particular, Font *et al* (2005) reported in 30 patients treated with sequential mitoxantrone plus prednisone followed by every 3 weeks docetaxel plus estramustine, a PSA response rate of 23% after mitoxantrone/prednisone with an increase to 63% after docetaxel/estramustine. The median progression-free survival was 10 months with a median survival of 18 months and the most frequent toxicity was grade 3–4 neutropaenia (13% of patients treated with mitoxantrone/prednisone and 20% with docetaxel/estramustine). In our sequential study, mitoxantrone did not contribute to increase overall schedule activity, but it seemed to be able to control advanced prostate cancer without relevant toxicity. An adequate trial incorporating time to progression or time to treatment failure as primary end point could better evaluate this chemotherapeutic strategy.

In conclusion, sequential chemotherapy with docetaxel plus estramustine followed by mitoxantrone plus prednisone is overall a well-tolerated regimen. However, although sequential use of mitoxantrone does not seem to improve docetaxel activity, its potential role as a maintenance therapy could be of interest for further studies in HRPC.

## Figures and Tables

**Table 1:** Patient characteristics

	**No (%)**
No. of patients	40
	
*Age*	
Median	72
Range	55–83
	
*ECOG performance status*	
0	19 (47.5)
1	21 (52.5)
Locally advanced disease	3 (8)
Metastatic disease	37 (92)
	
*Sites of disease*	
Prostate	27 (68)
Bone	27 (68)
Nodes	17 (43)
Liver	3 (8)
	
*No. of involved organs*	
1	11 (28)
>1	29 (73)
	
*Prior treatment*	
Hormone therapy	40 (100)
Radiotherapy	13 (33)
Prostatectomy	13 (33)
	
*Serum PSA*	
Median (ng ml^−1^)	58
Range	12–912
	
*Gleason score*	
⩽7	13 (32.5)
8–10	18 (45)
Not available	9 (22.5)
	
*Pain*	
VAS scale ⩾1	11 (27.5)

Abbreviation: ECOG=Eastern Cooperative Oncology Group.

**Table 2:** Maximum toxicity per patient

	**Docetaxel/estramustine (40 patients)**		**Mitoxantrone/prednisone (27 patients)**	
	**G1–2**	**G3–4**	**G1–2**	**G3–4**
**Toxicity**	**No. (%)**	**No. (%)**	**No. (%)**	**No. (%)**
Neutropaenia	4 (10)	1 (2.5)	9 (33)	2 (7)
Thrombocytopaenia	3 (7.5)	1 (2.5)	3 (11)	0 (0)
Anaemia	36 (90)	1 (2.5)	22 (81)	0 (0)
Nausea	28 (70)	1 (2.5)	5 (18.5)	0 (0)
Vomiting	20 (50)	0 (0)	2 (7)	0 (0)
Asthenia	29 (73)	2 (5)	16 (59)	1 (4)
Anorexia	25 (63)	0 (0)	9 (33)	0 (0)
Diarrhoea	24 (60)	0 (0)	7 (26)	0 (0)
Onycholysis	9 (22.5)	1 (2.5)	8 (29)	3 (11)
Dermatitis	9 (22.5)	1 (2.5)	4 (15)	0 (0)
Sensory neuropathy	8 (20)	0 (0)	0 (0)	0 (0)
Liver toxicity	8 (20)	7 (17.5)	1 (4)	0 (0)
Stomatitis	19 (47.5)	2 (5)	8 (29)	0 (0)
Deep venous thrombosis	0 (0)	4 (10)	0 (0)	1 (4)
Congestive heart failure	0 (0)	0 (0)	0 (0)	2 (7)

**Table 3:** PSA and objective response (RECIST)

	**No.**	**(%)**
*PSA response (39 patients)*		
PSA decline ⩾90%	15	37.5
95% CI		20–51
PSA decline ⩾50%	29	72.5
95% CI		63–82
PSA stabilisation	7	17.5
PSA progression	3	7.5
		
*Objective response (17 patients)*		
CR	2	12
PR	6	35
SD	3	17
CR+PR	8	47
95% CI		29–65
PD	6	35
